# Introduction to the Proceedings of the Avian Genomics and Gene Ontology Annotation Workshop

**DOI:** 10.1186/1471-2164-10-S2-I1

**Published:** 2009-07-14

**Authors:** Susan M Bridges, Shane C Burgess, Fiona M McCarthy

**Affiliations:** 1 Department of Computer Science and Engineering, Box 9637, Mississippi State University, Mississippi State, MS 39762, USA; 2 Department of Basic Sciences, College of Veterinary Medicine, Mississippi State University, Mississippi State, MS 39762, USA; 3 Institute for Digital Biology, Mississippi State University, Mississippi State, MS 39762; 4 Life Science and Biotechnology Institute, Mississippi State University, Mississippi State, MS 39762, USA

## Abstract

The Avian Genomics Conference and Gene Ontology Annotation Workshop brought together researchers and students from around the world to present their latest research addressing the delivery of value from the billions of base-pairs of Archosaur sequence that have become available in the last few years. This editorial describes the conference itself and introduces the ten peer-reviewed manuscripts accepted for publications in the proceedings. These manuscripts address issues ranging from the poultry industry view of USDA genomics policy to the genomics of a wide variety of Archeosaur species including chicken, duck, alligator, and condors and their pathogens.

## Introduction

On May 19–20, 2008 the Mississippi State University Institute for Digital Biology hosted an international meeting entitled *Delivering Value from Avian Genomes*. The meeting was predicated on the fundamental need to deliver valuable knowledge from the investment in omic biology in avian species. In this respect it was preceded by the *Blueprint for USDA Efforts in Agricultural Animal Genomics *2008–2017 [1] and by optimism from chicken production industries. Specific impetuses for this meeting were the aforementioned *Blueprint*, the sequencing of the zebra finch [2] and turkey genomes [3] and the US National Institute for Health's recognition of the importance of avian species in human disease and for biomedical research http://www.nih.gov/science/models/gallus/.

This meeting was the fourth international meeting with a focus on chicken genomics. It followed the *International Chicken Genome Workshop *(Hinxton Genome Campus, Cambridge, UK, March 10–11, 2003) held just prior to sequencing; the *International Meeting on the Chicken Genome *(Stowers Institute of Medical Research, Kansas City, Mo, USA, April 29 to May 2, 2004) which focused on the initial examination of the released chicken genome sequence; the two *Workshops on Chicken Genomics & Development *(Cold Spring Harbor Laboratories, NY, USA, May 8–11, 2005 and May 7 – 10, 2006) which focused on the first genome-scale experimental work enabled by the genome sequence; and the *International Chick Meeting *(11–14 April 2007, Barcelona, Spain) with a major focus on genomics and development, the immune system and evolutionary biology.

The genomics meeting at Mississippi State University built on these previous meetings but focused on deriving value from the investments in genomics and functional genomics in the chicken as well as other avian genomics research. It was also the first such meeting to focus also on "avian" genome sequences other than chicken. From the manuscripts that follow it is clear that the combination of second generation sequencing technologies and the ability to leverage the investment in the chicken genome sequence has enabled Archosaur genomics (the diapsid reptiles represented by modern birds and crocodilians and including extinct non-avian dinosaurs, pterosaurs and relatives of crocodiles). It is also notable that, although the chicken genome "was sequenced" by 2004 [4], it still requires structural annotation, more targeted sequencing to elaborate specific areas, experimental validation and sequencing of the gene-rich micro-chromosomes that currently are not included in the genome sequence at all. It is expected that efforts to sequence the turkey and the zebra finch will not only benefit from the chicken genome but that these species will, in turn, enhance the chicken genome sequence annotation. It is also likely that second (and later)-generation sequencing technologies will help fill in our knowledge gaps in the next few years.

Concurrently with technology advances in genome sequencing there is clearly an understanding in the avian research communities that, with enormous increases in genomic data, we need to ensure that we do not lose sight of the fundamental scientific questions. With this in mind there has been an increase in specific practical, accessible and user-friendly computational resources and tools dedicated to avian omic-scale biology; these will be essential if the goals of the *Blueprint for USDA Efforts in Agricultural Animal Genomics *2008–2017 [1] are to be achieved and for our avian models to fulfil their potential as biomedical research tools. The manuscripts in this BMC Genomics special supplement are those that were submitted, refereed and accepted for publication after this meeting. What is clear from these articles is the range and depth of the value that can be created now that the fundamental data source, along with computational tools built in the last five years, are available and democratizing the use this data.

## Proceedings summary

The Avian Genomics Conference was organized by Susan M. Bridges and Shane C. Burgess. Members of the program committee were Parker Antin, University of Arizona, David Burt, Roslin Institute, Edinburgh, Scotland, Youping Deng, University of Southern Mississippi, Mark Johnson, U.S. Army Center for Health Promotion and Preventative Medicine, Fiona McCarthy, Mississippi State University, Edward Perkins, U. S. Army Corps of Engineers Environmental Laboratory, and Carl Schmidt, University of Delaware. The Avian Gene Ontology (GO) Annotation Workshop was organized by Fiona M. McCarthy of Mississippi State University and members of the program committee were: Rama Balakrishana, Stanford University, Jennifer Clark, European Bioinformatics Institute, UK, Judith Blake, Jackson Laboratories, and Harold Drabkin, Jackson Laboratories.

The keynote speaker for the Avian Genomics Conference, Dr. Scott Edwards from Harvard University is internationally known for his ground-breaking research in the genomics based phylogenetics of many different avian species and reptiles including dinosaurs. One of the founders of the Gene Ontology (GO), Judith Blake of Jackson, Laboratories was the plenary speaker for the Avian GO Annotation Workshop.

A student poster competition was conducted and a panel of judges selected award winners. First place was awarded to Laura Lleras, King's College London, second place to Ang Li, University of Southern California, and third place tie to Catalina Tudor of the University of Delaware and Shyamesh Kumar of Mississippi State University.

Papers submitted for inclusion in the proceedings were peer-reviewed by two or more program committee members and other research area experts. The accepted papers demonstrate the breadth of research enabled by the availability of avian genomes. Papers have been grouped into several categories described in the following sections.

## Poultry genomics

Janet Fulton [5] of Hy-Line International reviews the *Blueprint for USDA Efforts in Agricultural Animal Genomics 2008–2017 *[1] from a poultry industry perspective with a focus on the tools, resources, and technologies specifically required for chicken. Fulton contends that the *Blueprint *has a significant bias toward the cattle industry and highlights areas where approaches in the cattle industry such as SNP analysis do not have direct applicability for poultry. She provides an extensive discussion of infrastructure needs for poultry research ranging from animal resource populations to bioinformatics tools.

Behnam Abasht and colleagues [6] analyze characteristics required for Marker Assisted Selection (MAS) in commercial layer chicken populations. Effective MAS requires high linkage disequilibrium (LD) between markers and quantitative trail loci (QTL) and sustained marker-QTL LD over generations. Their study uses two different methods to assess the level and consistency of LD between SNPs to identify markers associated with egg-quality and egg-production phenotypes. Their results indicate that markers will retain high LD with linked QTL and be effective for MAS.

Tamsyn Crowley and colleagues [7] investigate the utility of whole genome chicken microarrays for cross-species analysis of other avian species including duck. They demonstrate that cross species hybridization (CSH) provides reliable signals and that whole genome long oligonucleotide chicken microarrays provide a valuable resource for studying gene expression in a range of avian species.

Bindu Nanduri and colleagues [8] study the transcriptional response of the gram-negative bacterial pathogen *Pasteurella multiocida*, the agent of fowl cholera in poultry, to sub-lethal doses of three different classes of antibiotics. They demonstrate common adaptive responses to antibiotic stress and also identify differences in the responses. Their systems biology analysis demonstrates both the possibilities and the challenges of modelling high throughput datasets in non-model avian bacteria.

## Bioinformatics resources

The utility of the chicken and other avian genomes critically depends on the development of appropriate and specialized bioinformatics tools. Consistent and meaningful gene nomenclature fundamentally underpins bioinformatics analysis and David Burt and colleagues [9] report the formation of the Chicken Gene Nomenclature Committee (CGNC). The CGNC, in collaboration with the research community as a whole, will provide standardized nomenclature for the chicken genome that reliably links chicken genes across all databases follow nomenclature and associated guidelines established by the Human Genome Nomenclature Committee (HGNC).

Effective application of systems biology to emerging model species including avians requires extensive interaction data that are often unavailable. Jay Konieczka and colleagues [10] describe the open-source BioNetBuilder2.0 client-server Cytoscape [11] plugin that automatically integrates molecular interactions from all major public interaction databases and provides these directly to the user's Cytoscape environment. The article includes a detailed tutorial of all steps required in the analysis.

Using aquaporins as a case study, Raphael Isokpehi and colleagues [12] investigate how the integration of chicken and mammalian sequence, as well as gene expression resources, enable functional genomics research in both avian and mammalian species. To do so they demonstrate the types of hypotheses that can be generated by integrating a wide variety of on-line resources.

## Archeosaur genomics

Previous international conferences dealing with avian genomics had focused almost exclusively on chicken. In contrast, the scope of *Delivering Value from Avian Genomes *was consciously expanded to include all avian species and related archosaurs such as crocodilians, lizards, and dinosaurs. Chapus and Edwards [13] use paired bacterial artificial chromosome (BAC) end sequences from the American alligator, painted turtle, emu and chicken to investigate patterns of sequence divergence, gene and retroelement content and microsynteny in Reptilia. Their results provide insights into the evolution of the lineage leading to chicken including the drastic reduction in genome size observed in birds.

Shan and colleagues [14] describe the development of a high quality BAC library for a second crocodilian, the Australian saltwater crocodile. They demonstrate the utility of the new resource for gene isolation, genome characterization, and comparative genomics.

Romanov and colleagues [15] demonstrate the application of genomic studies of wild birds to comprehensive conservation including deeper understanding of mechanisms affecting genetic variation, adaptation, and evolution. They provide case studies of genotyping a California condor resource population to provide an improved assessment of current population genetic variation and also genomic studies of the white-throated sparrows to understand evolution of natural populations.

## Competing interests

The authors declare that they have no competing interests.

## Authors' contributions

All authors served as co-editors of these proceedings and all authors helped write this introduction.
